# Activation of α_1A_-adrenergic receptor promotes differentiation of rat-1 fibroblasts to a smooth muscle-like phenotype

**DOI:** 10.1186/1471-2121-5-47

**Published:** 2004-12-16

**Authors:** Abdelwahab E Saeed, Jean-Hugues Parmentier, Kafait U Malik

**Affiliations:** 1 Department of Pharmacology, College of Medicine, The University of Tennessee Health Science Center, 874 Union Avenue, Memphis, TN 38163, USA

## Abstract

**Background:**

Fibroblasts, as connective tissue cells, are able to transform into another cell type including smooth muscle cells. α_1A_-adrenergic receptor (α_1A_-AR) stimulation in rat-1 fibroblasts is coupled to cAMP production. However, the significance of an increase in cAMP produced by α_1A_-AR stimulation on proliferation, hypertrophy and differentiation in these cells is not known.

**Results:**

Activation of the α_1A_-AR in rat-1 fibroblasts by phenylephrine (PE) inhibited DNA synthesis by 67% and blocked the re-entry of 81% of the cells into S phase of the cell cycle. This cell cycle blockage was associated with hypertrophy characterized by an increase in protein synthesis (64%) and cell size. Elevation of cAMP levels decreased both DNA and protein synthesis. Inhibition of adenylyl cyclase or protein kinase A reversed the antiproliferative effect of cAMP analogs but not PE; the hypertrophic effect of PE was also not altered. The functional response of rat-1 cells to PE was accompanied by increased expression of cyclin-dependent kinase (Cdk) inhibitors p27^kip1 ^and p21^cip1/waf1^, which function as negative regulators of the cell cycle. Stimulation of α_1A_-AR also upregulated the cell cycle regulatory proteins pRb, cyclin D1, Cdk 2, Cdk 4, and proliferating cell nuclear antigen. The antiproliferative effect of PE was blocked by p27^kip1 ^antisense but not sense oligonucleotide. PE also promoted expression of smooth muscle cell differentiation markers (smooth muscle alpha actin, caldesmon, and myosin heavy chain) as well as the muscle development marker MyoD.

**Conclusions:**

Stimulation of α_1A_-AR promotes cell cycle arrest, hypertrophy and differentiation of rat-1 fibroblasts into smooth muscle-like cells and expression of negative cell cycle regulators by a mechanism independent of the cAMP/PKA signaling pathway.

## Background

Alpha1-adrenergic receptors (α_1_-ARs) are members of the G-protein-coupled receptor superfamily. Both pharmacological and molecular cloning studies have indicated the existence of multiple subtypes of α_1_-ARs including α_1A/C_-AR, α_1B_-AR, and α_1D_-AR [1-4]. α_1_-ARs play a key role in a variety of physiological processes, such as contraction of vascular and cardiac muscle, contraction of the spleen, liver glycogenesis, or melatonin secretion in the pineal gland [3,4]. Activation of α_1A_-AR promotes hypertrophy of cardiac myocytes [5,6]. Recently it has been shown that all three subtypes of α_1_-AR are also expressed in rat aortic adventitial fibroblasts and vascular smooth muscle cells (SMC) [7] and their activation with norepinephrine stimulates migration, proliferation and protein synthesis [8,9]. However, norepinephrine increased SMC hypertrophy, but not DNA synthesis, through α_1A_-AR stimulation in uninjured aorta whereas norepinephrine stimulated proliferation of adventitial fibroblasts through the α_1B_-AR subtype [8].

Nonvascular fibroblasts, including cardiac fibroblasts [7,10], generally do not express α_1_-AR and have been used for stable transfection of different subtypes of α_1_-AR to study their respective functions. However, a recent study showed the expression of a functional α_1A_-AR in primary tendon fibroblasts [11]. In rat-1 cells, a transformed cell line from embryonic fibroblast, expressing different subtypes of α_1_-AR, phenylephrine (PE), an α_1_-AR agonist, activates phospholipase D and releases arachidonic acid [12]. However, unlike SMC, activation of α_1_-ARs in rat-1 cells also increases cAMP levels and PKA activity [12]. α_1A_-AR is more efficiently coupled to phospholipase D activation, arachidonic acid release and cAMP than α_1B_-AR or α_1D_-AR in these cells [12]. Activation of α_1A_-AR expressed in COS-7 and HeLa cells [13] and α_1B_-AR or α_1D_-AR in COS and CHO cells [14] also increase cAMP levels. In HepG2 and M12 cells expressing α_1B_-AR, PE causes cell scattering and inhibits proliferation through activation of MAP kinases [15].

The family of connective tissue cells includes fibroblasts, cartilage cells, bone cells, fat cells and smooth muscle cells. Fibroblasts seem to be able to transform into any of other members of the family – in some cases reversibly – although it is not clear whether this is a property of a single type of fibroblast that is pluripotent or of a mixture of distinct types of fibroblasts with more restricted potentials. These transformations of connective tissue cell type are regulated by the composition of the surrounding extracellular matrix, by cell shape, and by hormones and growth factors [16]. Heterologous expression of α_1A_-ARs in CHO cells inhibits basal and growth factor-stimulated DNA synthesis, in contrast to the α_1D_-AR [17]. A recent study in the same model has reported cAMP as the mediator of the antiproliferative effect of α_1A_-AR stimulation [18]. Therefore, it is possible that activation of α_1A_-AR with PE in rat-1 cells affects their growth and/or differentiation status. To test this hypothesis, we have investigated the effect of PE and cAMP modulators on proliferation, growth and morphology in rat-1 cells expressing α_1A_-ARs. Moreover, we have examined the effect of PE and cAMP modulators on the expression of cell cycle regulators and muscle cell markers, because of the ability of fibroblasts to differentiate into myofibroblasts. Our results show that activation of α_1A_-ARs in rat-1 cells exerts profound effects promoting hypertrophy and expression of specific smooth muscle cell markers. We also show here that α_1A_-AR-induced cessation of DNA synthesis is independent of cAMP and involves the expression of cyclin-dependent kinase (Cdk) inhibitor, p27^kip1^.

## Results

### Stimulation of α_1A_-AR inhibits DNA synthesis at the G_1_/S checkpoint of the cell cycle in rat-1 fibroblasts

**Figure 1:**
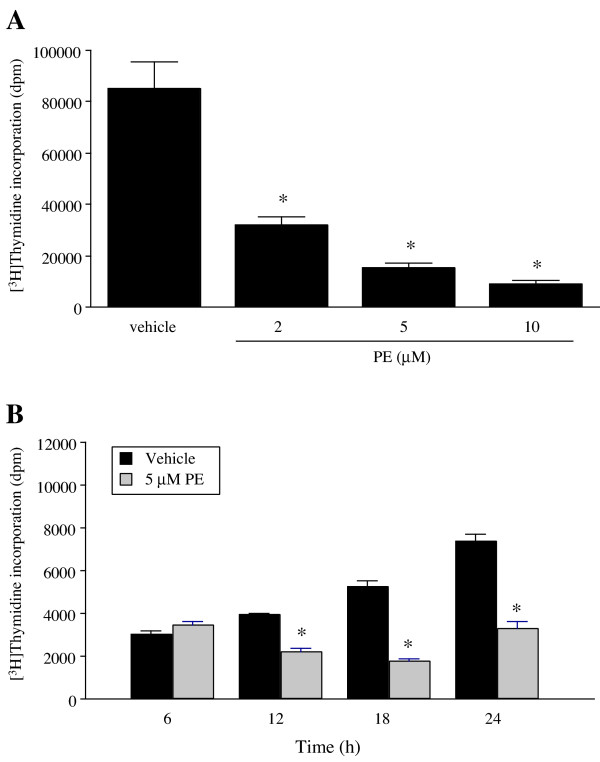
Effect of PE on [^3^H]thymidine incorporation in rat-1 cells. Cells were grown in 24-well plates in DMEM containing 10% FBS at a density of 150,000 cells/well until they reached 80–90% confluency, and then serum-deprived for 48 h in DMEM. A, Cells were treated with different concentrations of PE (2, 5, and 10 μM) for 18 h prior to the addition of [^3^H]thymidine (0.50 μCi/ml) for the last 4 h. B, Cells were treated with PE for different incubation periods prior to the addition of [^3^H]thymidine (0.50 μCi/ml). Cells were incubated with [^3^H]thymidine for the last 4 h and thymidine incorporation was determined as described in Methods. Data are expressed as dpm of [^3^H]thymidine incorporated in DNA per well. Values are the mean ± S.E. of six independent experiments performed in quadruplicates on different batches of cells. * Values significantly different from vehicle, p < .05.

Rat-1 fibroblasts stably transfected with α_1A_-AR expressed 288 ± 2 fmol/mg protein of receptors [12]. Cells at 80% confluency were serum-deprived for 48 h in DMEM and then incubated with PE (2, 5 and 10 μM) for different periods prior to the addition of [^3^H]thymidine. PE decreased basal [^3^H]thymidine incorporation in a concentration-dependent manner with a maximum effect at 10 μM PE (Fig 1A). Reduction of basal DNA synthesis was significant after 12 h of treatment with a maximum effect at 18 h of incubation with PE (> 65% decrease) (Fig 1B). Rat-1 fibroblasts stably transfected with α_1A_-AR incorporate [^3^H]thymidine in DNA in a time-dependent manner (from 6 to 24 h), even after 48 h of serum-deprivation (Fig 1B).

To determine whether PE at different concentrations delays the re-entry of rat-1 cells into the cell cycle, we examined the kinetics of cell cycle re-entry. Flow cytometry studies showed that incubation of the rat-1 cells with 2, 5, and 10 μM PE resulted in inhibition of the re-entry of 83, 81, or 85%, respectively, of the cell population into the S phase of the cell cycle (Table 1). Therefore, stimulation of α_1A_-AR inhibits DNA synthesis at the G_1_/S checkpoint in rat-1 fibroblasts.

**Table 1:** Effect of PE on cell cycle phases.

**Treatment**	**G_0_/G_1 _phase**	**S phase**	**G_2_/M phase**	**% inhibition (S)**
vehicle	9269 ± 0.5	319.5 ± 13.5	238.5 ± 23.5	0 %
2 μM PE	9770 ± 9.0	54.0 ± 2.0	125 ± 21.0	83%
5 μM PE	9699 ± 29.0	59.0 ± 4.0	170 ± 11.0	81%
10 μM PE	9761.5 ± 89.5	47.5 ± 13.5	110.5 ± 39.5	85%

Cells were subjected to flow cytometric DNA analysis as described in Methods. Different concentrations of PE inhibited the re-entry of the cell population into S phase of the cell cycle. Data show the number of cells in each phase of the cell cycle. Values are the mean ± S.E. of three independent experiments performed in duplicate on different batches of cells (Total events per cycle is 10,000 cells).

### Stimulation of α_1A_-AR increases protein synthesis and promotes hypertrophy in rat-1 fibroblasts

**Figure 2:**
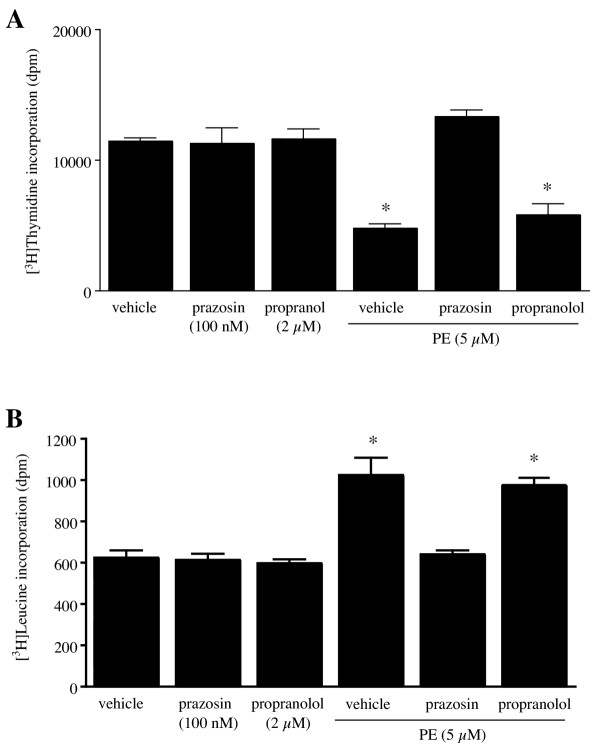
**Stimulation of α_1A_-AR mediates the effect of PE on DNA and protein synthesis. **A, Effect of prazosin (α_1_-AR antagonist) and propranolol (β-AR antagonist) on PE-induced inhibition of DNA synthesis. Cells were treated with prazosin and/or propranolol for 30 min before the addition of 5 μM PE for 18 h. [^3^H]thymidine incorporation was measured as described in Methods. Data are expressed as dpm of [^3^H]thymidine incorporated per well. Values are the mean ± S.E. of three independent experiments performed in quadruplicates on different batches of cells. * Value significantly different from the corresponding value obtained without PE treatment, p < .05. B, Effect of prazosin and propranolol on PE-induced increase in protein synthesis. Experimental conditions were similar as described in A. [^3^H]leucine incorporation was measured as described in Methods. Data are expressed as dpm of [^3^H]leucine incorporated per well. Values are the mean ± S.E of three independent experiments performed in quadruplicates on different batches of cells. * Value significantly different from the corresponding value obtained without PE treatment, p < .05.

Stimulation of α_1_-ARs in vascular smooth muscle cells [19] and in adult rat ventricular myocytes [8] increases hypertrophy and protein synthesis. However, the α_1_-AR subtype mediating these effects is not known. Exposure to 5 μM PE for 18 h increased [^3^H]leucine incorporation by 64% (Fig 2B) and decreased basal [^3^H]thymidine uptake (Fig 2A), as shown earlier in Fig 1. These effects were blocked by prazosin (PRZ, 100 nM), an α_1_-AR antagonist. Propranolol (2 μM), a β-AR antagonist (Fig 2A,B), or yohimbine (1 μM), an α_2_-AR antagonist (data not shown), had no effect on the decrease in basal [^3^H]thymidine or the increase in [^3^H]leucine incorporation in rat-1 cells caused by exposure to PE. These results show that PE stimulates protein synthesis and inhibits DNA synthesis in rat-1 cells through activation of α_1A_-ARs. The hypertrophy index, an indicator of cellular hypertrophy, is defined as the ratio of protein synthesis ([^3^H]leucine incorporation) to DNA synthesis ([^3^H]thymidine incorporation) [20]. At 18 h, this ratio was 4.4 fold as high in PE-treated cells (index = 0.22) than in control cells (index = 0.05, n = 6), indicating that PE promotes rat-1 cell hypertrophy.

**Figure 3:**
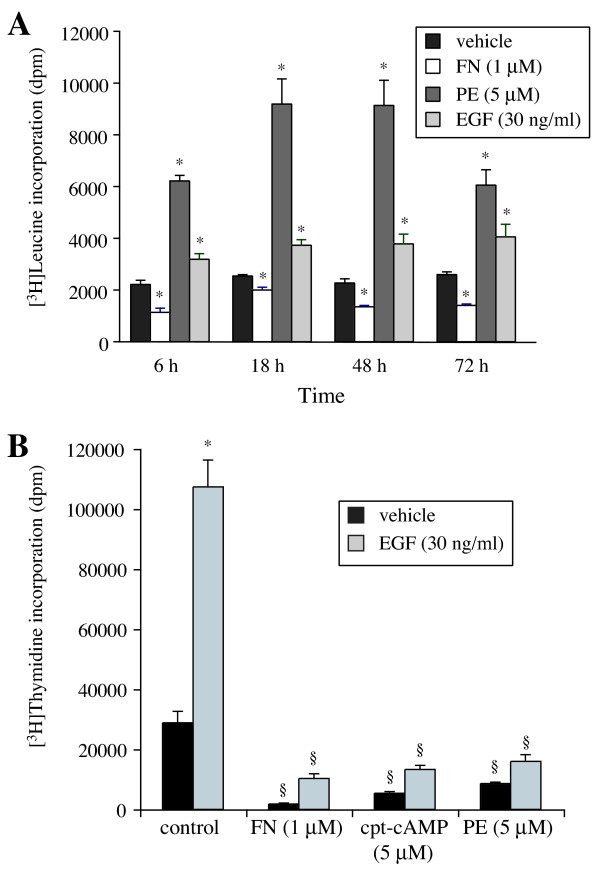
**Effect of PE, cAMP-elevating agents and EGF on protein and DNA synthesis**. A, Cells were treated with 5 μM PE, 1 μM forskolin (FN) and 30 ng/ml EGF for different periods. [^3^H]leucine incorporation was measured as described in Methods. Data are expressed as dpm of [^3^H]leucine incorporated per well. Values are the mean ± S.E. of three independent experiments performed in sextuplicates. * Values significantly different from vehicle, p < .05. B, Effect of FN, 8-cpt-cAMP and PE on EGF-induced [^3^H]thymidine incorporation. Cells were preincubated with FN (1 μM), 8-cpt-cAMP (5 μM) and PE (5 μM) for 1 h. Cells were then treated with EGF (30 ng/ml) for 18 h. Incorporation of [^3^H]thymidine into rat-1 fibroblasts was determined as described in Methods. Data are expressed as dpm of [^3^H]thymidine incorporated per well. Values are the mean ± S.E. of three independent experiments performed in duplicate in different batches of cells. * Value significantly different from vehicle, p < .05. § Value significantly different from the corresponding value obtained without treatment by FN, cpt-cAMP or PE, p < .05.

Protein synthesis elicited by α_1_-AR stimulation was time-dependent and remained significant from 6 to 72 h of exposure with PE (Fig 3A). EGF and cAMP have opposite effects on rat-1 fibroblasts proliferation [21,22]. Therefore, we tested the effect of EGF and forskolin (FN), an activator of adenylyl cyclase [12], on protein and DNA synthesis. EGF (30 ng/ml) slightly increased, whereas FN decreased, [^3^H]leucine incorporation at 6, 18, 48 and 72 h (Fig 3A). Surprisingly, PE was a better activator of protein synthesis than EGF. In contrast, EGF increased [^3^H]thymidine incorporation by 272% (Fig 3B), an effect blocked by FN and the cAMP analog, 8-cpt-cAMP, as well as by PE.

### PE inhibits DNA synthesis and promotes hypertrophy by a mechanism independent of cAMP

**Figure 4:**
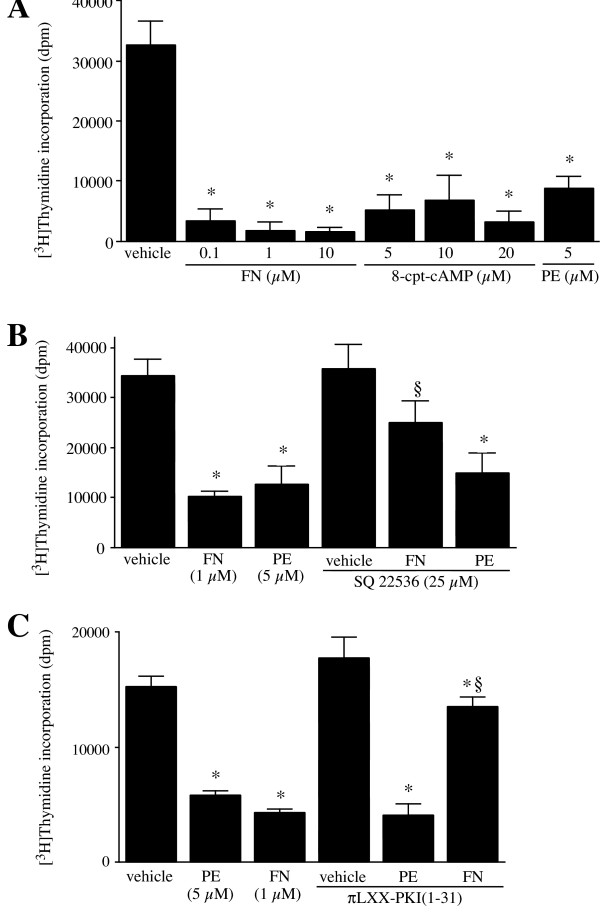
**Effect of PE and cAMP-elevating agents, FN and 8-cpt-cAMP, on DNA synthesis**. Cells were serum-deprived for 2 days. A, Cells were treated with PE (5 μM), FN (0.1, 1, and 10 μM), and 8-cpt-cAMP (5, 10, and 20 μM) for 18 h. Incorporation of [^3^H]thymidine into rat-1 fibroblasts was determined as described in Methods. Data are expressed as dpm of [^3^H]thymidine incorporated per well. Values are the mean ± S.E. of three independent experiments performed in sextuplicates in different batches of cells. * Value significantly different from vehicle, p < .05. B, Effect of inhibitors of adenylyl cyclase inhibition on PE and cAMP-elevating agents. Cells were pre-incubated with SQ22536 (25 μM), an specific adenylyl cyclase inhibitor for 1 h. Cells were then treated with 5 μM PE and/or 1 μM FN for 18 h. [^3^H]thymidine incorporation was determined as described in Methods. Values are the mean ± S.E. of three independent experiments performed in quadruplicates on different batches of cells. Data are expressed as dpm of [^3^H]thymidine incorporated per well. * Value significantly different from the corresponding vehicle, p < .05. § value significantly different from that obtained in the presence of agonist alone, p < .05. C, Effect of transfection of πLXX-PKI [1-31] on PE-induced inhibition of DNA synthesis. Preconfluent cells were transiently transfected with πLXX-PKI [1-31], a plasmid encoding for the protein kinase inhibitor, (PKI), using Lipofectamine Plus transfection reagent according to the manufacturer's protocol (Life Technologies). Cells were cultured in serum-free DMEM for 24 h and then treated with 5 μM PE and/or 1 μM FN. Cells were assayed for [^3^H]thymidine incorporation 48 h post transfection as described in Methods. Data are expressed as dpm of [^3^H]thymidine incorporated per well. Values are the mean ± S.E. of three independent experiments performed on different batches of cells. * Value significantly different from the corresponding vehicle, p < .05. § value significantly different from that obtained in the presence of agonist alone, p < .05.

Previously, we have shown that PE induces cAMP accumulation in rat-1 cells [12]. It is also known that cAMP-dependent PKA-mediated inhibition of cell division is due to blockade of growth factor-stimulated cell cycle progression from G_1 _to S phase [23]. Therefore, to determine whether cAMP mediates the antiproliferative effect of PE, we examined the effect of FN (0.1–10 μM) and 8-cpt-cAMP (5–20 μM) and of FN and PE on DNA synthesis in the presence and absence of adenylyl cyclase and PKA inhibitors. FN and 8-cpt-cAMP inhibited [^3^H]thymidine incorporation to the same extent as PE (Fig 4A). The FN but not the PE-induced decrease in [^3^H]thymidine incorporation was blunted by the selective inhibitor of adenylyl cyclase SQ 22536 (Fig 4B) and by the P-site inhibitors of adenylyl cyclase 2',5'dideoxyadenosine, and 2'-deoxyadenosine 3'-monophosphate (5 μM each-data not shown). The selectivity of cAMP modulators, FN, 8-cpt-cAMP and SQ 22536 has been tested previously in rat-1 fibroblasts [12]. To further determine the contribution of cAMP pathway, rat-1 cells were transfected with πLXX-PKI [1-31], a vector that expresses the PKA inhibitor, PKI [23,24]. Transfection with PKI reversed the inhibitory effect of FN, but not that of PE, on [^3^H]thymidine incorporation in rat-1 cells (Fig 4C). These observations indicate that cAMP and PKA do not mediate the PE-induced inhibition of DNA synthesis.

The antiproliferative effect of PE, FN and 8-cpt-cAMP could be the result of cell loss due to a cytotoxicity. However, the trypan blue exclusion test and LDH assay showed that more than 90% of cells were viable (Table 2) and less than 1% of the cells were found in the supernatant, indicating very little cell loss.

**Table 2:** Effect of PE and cAMP elevating agents on viability of rat-1 fibroblasts using the hemocytometer trypan blue method.

**Treatment**	**Total number of cells**	**Blue cells**	**% of viability**
Vehicle	78	8	90%
5 μM PE	142	10	92%
10 μM PE	87	12	86%
10 μM FN	78	8	90%
20 μM 8-cpt-cAMP	84	4	96%
20 μM 8-Br-cAMP	66	2	97%

Cells were incubated for 18 h with PE (5 and 10 μM), forskolin (FN), 8-cpt-cAMP and 8-Br-cAMP. The percentage of viable cells was calculated. Values are representative of five different experiments.

### Morphological change elicited by α_1A_-AR stimulation and by cAMP in rat-1 cells

**Figure 5:**
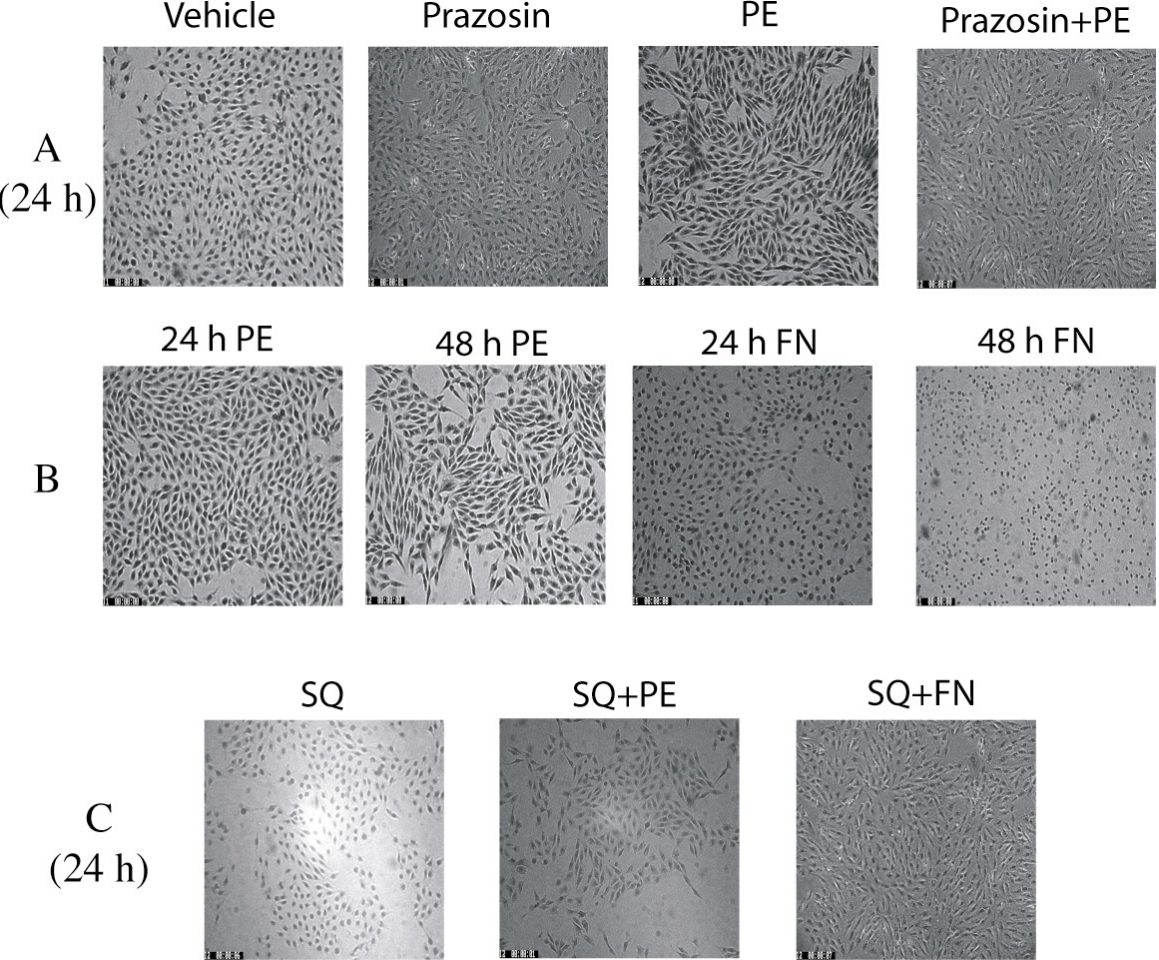
**Effect of prazosin and SQ22536 on PE-and FN-induced morphological change in rat-1 cells**. Preconfluent, serum-deprived cells were either stimulated with 5 μM PE and/or 1 μM FN for different periods of time (Fig 5B) and/or pretreated for 30 min with 100 nM prazosin (Fig 5A) or 25 μM SQ22536 (Fig 5C), and then further incubated with 5 μM PE and/or 1 μM FN for 24 h. Morphological studies were performed as described in Methods. The same morphological patterns were obtained in three different experiments.

Although both PE and FN inhibited DNA synthesis in rat-1 cells, they produced different effects on cell morphology. PE increased, whereas FN decreased, the apparent size of rat-1 cells as shown in Fig 5. Prazosin (100 nM), an α_1_-AR antagonist, reversed the shape change produced by PE. To determine if PE-induced cell cycle arrest and hypertrophy of rat-1 fibroblasts is also independent of cAMP, we preincubated the cells with SQ22536 (25 μM) and then treated them with PE (5 μM) or FN (1 μM) for different time periods. SQ22536 reversed the morphological changes produced by FN and restored the normal fibroblast shape, whereas it enhanced the effect of PE, i.e. the cells became more hypertrophic.

### p27^kip1 ^mediates PE-induced inhibition of DNA synthesis

**Figure 6:**
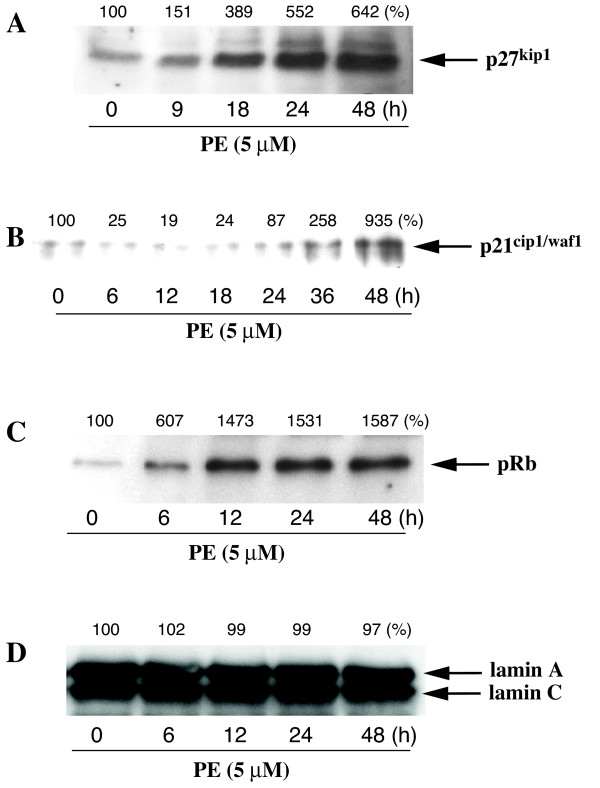
**Effect of PE on the expression of cell cycle regulators, p27^kip1^, p21^ip1/waf1 ^and pRB**. Preconfluent, serum-deprived cells were incubated with PE (5 μM) or vehicle for up to 48 h. Expression of cell cycle regulators was determined by Western blot analysis of whole cell lysates as described in Methods. The panels are representative Western blots showing the effect of PE on the expression of p27^kip1 ^(A), p21^cip1/waf1 ^(B), pRb (C), and lamin A/C (D) protein levels respectively. Percentages representing the quantitation of protein bands by densitometric analysis of blots obtained from three different experiments are indicated on top of each immunoblot.

Cyclin-dependent kinase (cdk) inhibitors p27^kip1 ^and p21^cip1/waf1 ^play an important role in cell-cycle regulation [25,26]. High levels of p27^kip1 ^and p21^cip1/waf1 ^in quiescent (G_0_) cells decline upon induction of mitogenesis [27]. This decrease in Cdk inhibitors appears to be critical in enabling cell cycle entry. Western blot analysis showed that α_1A_-AR stimulation increased the expression levels of p27^kip1 ^(642% of control at 48 h) and p21^cip1/waf1 ^(935% of control at 48 h) (Fig. 6A). The protein levels remained elevated for 48 h, with levels of p27^kip1 ^reaching 389% of control at 18 h of incubation and remaining elevated for up to 48 h. This increase was concomitant with the inhibitory effect of PE on DNA synthesis, which was also maximal at 18 h of incubation. PE increased the expression of p27^kip1 ^(552% of control at 24 h) more efficiently than p21^cip1/waf1^(87% of control at 24 h) suggesting a greater contribution of p27^kip1 ^to PE-induced cell cycle arrest and a delayed induction of p21^cip1/waf1^. Retinoblastoma protein (pRb) hyperphosphorylation and subsequent release of E2F is required for cell cycle progression [28]. However, α-AR stimulation of neonatal myocytes has been shown to induce pRb hyperphosphorylation without induction of cell proliferation [29]. Our results demonstrate that PE promotes an early increase in pRb levels (607% of control at 6 h) in rat-1 cells (Fig 6C), associated with a decrease in DNA synthesis (Fig 1B). The nuclear envelope proteins lamin A and C, used as control, were not altered by PE treatment (Fig 6D).

**Figure 7:**
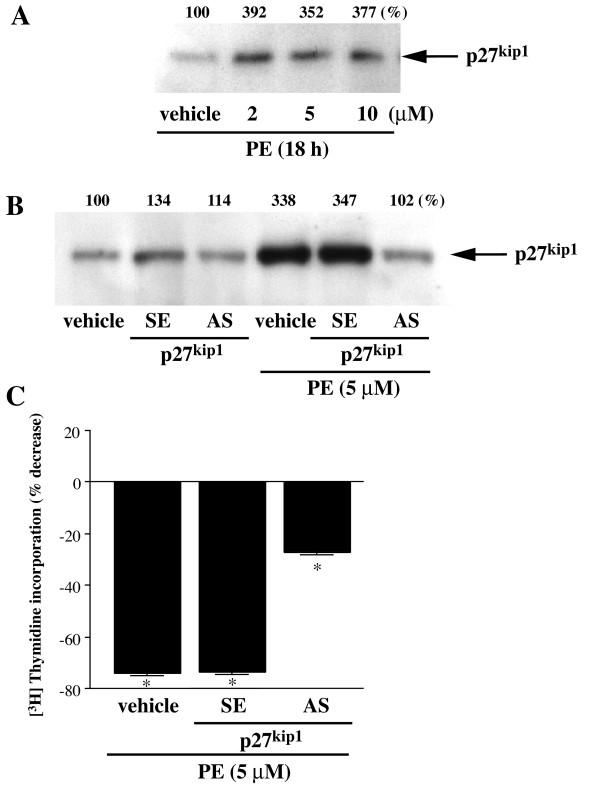
**Effect of PE on p27^kip1 ^level and effect of p27^kip1 ^antisense oligonucleotides on PE-induced inhibition of DNA synthesis**. A, Preconfluent rat-1 cells were stimulated with 2, 5, and 10 μM PE for 18 h. Total cell lysates were subjected to Western blot analysis as described in Methods. Percentages representing the quantitation of protein bands by densitometric analysis of blots obtained from three different experiments are indicated on top of each immunoblot. B, Representative Western blot showing the effect of p27^kip1 ^antisense and sense oligonucleotides treatment on PE-induced increase in p27^kip1 ^protein level. Cells were transiently transfected with p27^kip ^antisense or sense oligonucleotide for 48 h using Oligofectamine™ Reagent according to the manufacturer's instruction (Invitrogen). C, Transfected cells as in B were serum-starved for 24 h and then treated with 5 μM PE. Thymidine uptake was measured as described in Methods 48 h post-transfection. Data are expressed as the percent decrease in [^3^H]thymidine uptake above the basal value obtained in unstimulated cells. Values are the mean ± S.E of three independent experiments performed in different batches of cells. * Value significantly different from the corresponding vehicle, p < .05.

In VSMC, p27^kip1 ^level is a critical determinant of the cell response, hyperplasia vs. hypertrophy, to angiotensin II and other growth factors [20]. We postulated that the response of rat-1 cells to PE was mediated by p27^kip1^. PE upregulates the expression of p27^kip1 ^at 18 h of treatment in rat-1 cells at all concentrations (Fig 7A). We therefore explored further the role of p27^kip1 ^in modulating the antiproliferative response of rat-1 cells to PE by transfecting these cells with p27^kip1 ^antisense and sense ODN. p27^kip1 ^antisense (102% of control), but not sense ODN (347% of vehicle vs. 338% for PE alone), diminished p27^kip1 ^level (Fig 7B) and blocked the antiproliferative effect of PE in rat-1 cells (Fig 7C). These observations suggest that PE-induced inhibition of DNA synthesis is mediated by p27^kip1^.

### PE increases the expression of cyclin D1, proliferating cell nuclear antigen (PCNA), Cdk2 and Cdk4 in rat-1 cells

**Figure 8:**
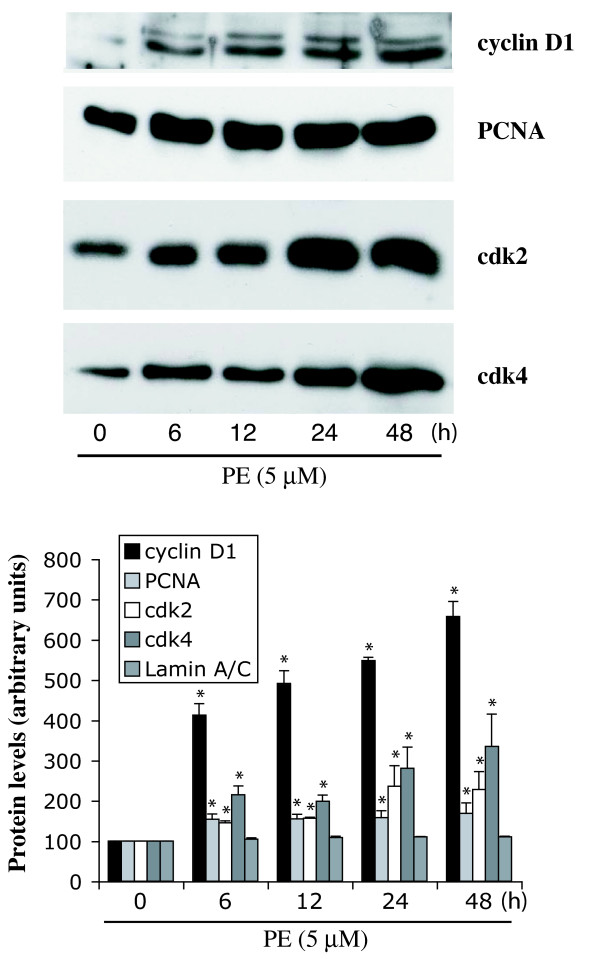
**Effect of PE on the expression of cyclin D1, PCNA Cdk 2, and Cdk 4**. Preconfluent, serum-deprived cells were stimulated with PE (5 μM) for 6, 12, 24 and 48 h. Cell-cycle regulators expression was determined by Western blot analysis of whole cell lysates as described in Methods. The bar graph represents quantitation of protein bands by densitometric analysis of blots obtained from three different experiments. * Value significantly different from the corresponding vehicle, p < .05.

We next investigated the effect of PE on several cell cycle proteins important for G_1_/S phase progression. Fig. 8 shows that treatment of serum-deprived rat-1 cells with PE increased the levels of cyclin D1 (548% of control at 24 h), PCNA (158% of control at 24 h), and Cdk2 (236% of control at 24 h) in a time-dependent manner. The levels of nuclear envelope proteins lamin A and C, were not altered. These results are unexpected since PE promotes cell cycle arrest at G_1_/S checkpoint (Table 1). However, the up-regulation of p27^kip1 ^seems to be sufficient to block cell cycle entry into S phase and DNA synthesis. Surprisingly, we also observed an increase in the protein levels of Cdk4 (280% of control at 24 h), which normally do not change even during mitogenic stimulation.

### PE increases expression of specific smooth muscle differentiation markers as well as MyoD

**Figure 9:**
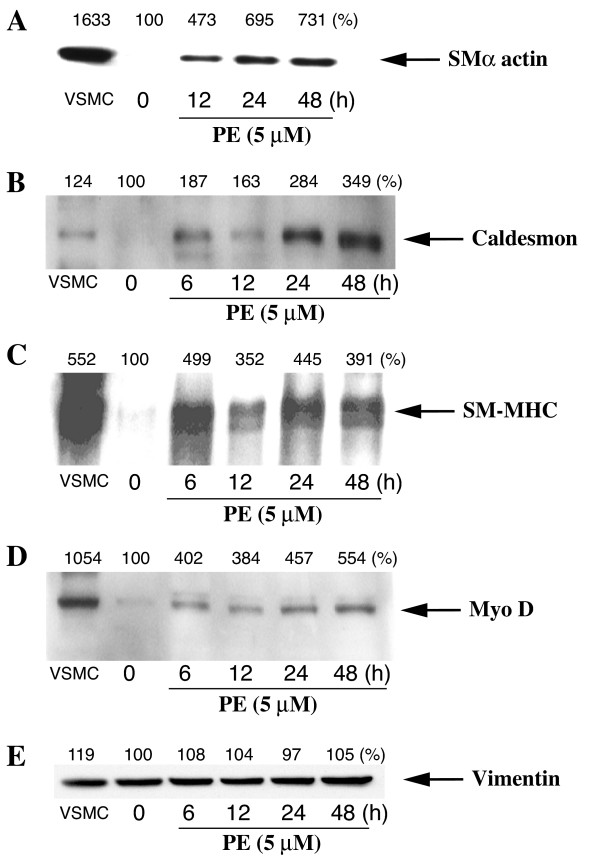
**Time course of the effect of PE on the expression of the smooth muscle differentiation markers and MyoD**. Preconfluent, serum-deprived fibroblasts were stimulated with PE (5 μM) for up to 48 h. The protein expression of smooth muscle differentiation markers and MyoD was determined by Western blot analysis of whole cell lysates as described in Methods. A, smooth muscle α actin (SMα actin). B, caldesmon. C, smooth muscle myosin heavy chain (SM-MHC). D, MyoD, E, vimentin. VSMC lysate is shown as a positive control. Percentages representing the quantitation of protein bands by densitometric analysis of blots obtained from three different experiments are indicated on top of each immunoblot.

**Figure 10:**
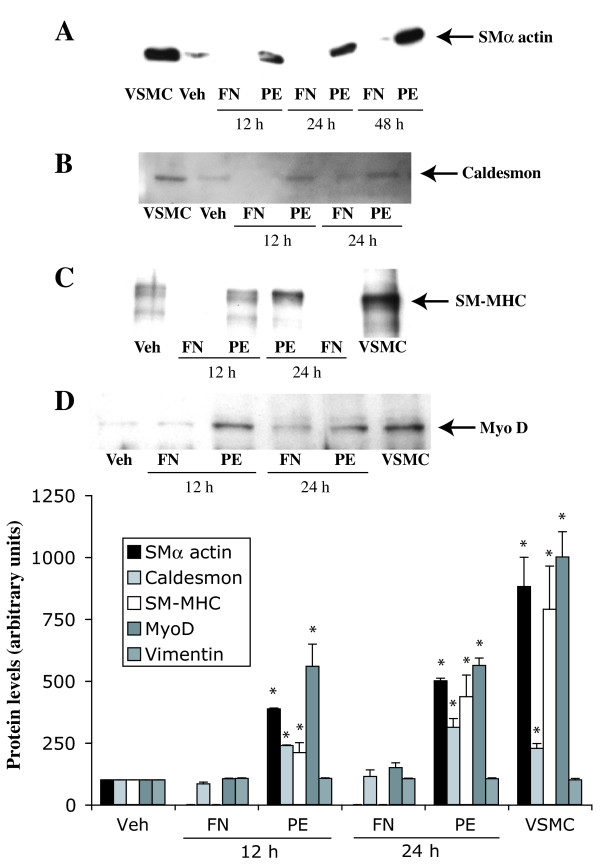
**Effect of PE and FN on the expression of smooth muscle differentiation markers and MyoD. **Preconfluent, serum-deprived fibroblasts were stimulated with either PE (5 μM) or FN (1 μM) for up to 48 h. Expression of markers was determined by Western blot analysis of whole cell lysates as described in Methods. A, smooth muscle α actin (SMα actin). B, caldesmon. C, smooth muscle myosin heavy chain (SM-MHC). D, MyoD. VSMC lysate is shown as a positive control in panel A, B, C and D. The bar graph represents quantitation of protein bands by densitometric analysis of blots obtained from three different experiments. * Value significantly higher than the corresponding vehicle, p < .05.

Cell cycle arrest is closely coupled to muscle cell differentiation and is required for activation of muscle cell specific gene expression [16]. Since stimulation of α_1A _ARs promoted a change in morphology associated with hypertrophy, we examined the effect of PE on expression of the specific smooth muscle cell markers, α-smooth muscle actin (SMα actin), caldesmon and myosin heavy chain (SM-MHC) [30]. The smooth muscle markers were not expressed in serum-deprived rat-1 cells (Fig 9A,C). Stimulation of α_1A _ARs with PE (5 μM) promoted a time-dependent increase in the levels of SMα actin (695% of control at 24 h) (Fig 9A), caldesmon (284% of control at 24 h) (Fig 9B) and SM-MHC (445% of control at 24 h) (Fig 9C) as shown by Western blot analysis We also investigated the expression of MyoD, a helix-loop-helix protein that plays an important role in the regulation of muscle development [31]. Western blot analysis showed that PE also increased the expression of MyoD in a time-dependent manner (457% of control at 24 h), further supporting the evidence that stimulation of α_1A _AR induces differentiation of rat-1 fibroblasts into muscle-like cells, which is closely coordinated with cell cycle arrest (Fig 9D). The expression of vimentin, an intermediate filament protein used as a control, was not altered by treatment with PE (Fig 9E). The α_1A _AR stimulation of smooth muscle differentiation markers was independent of cAMP because FN did not increase the expression of SMα actin, caldesmon or SM-MHC (Fig 10A,C). MyoD expression was not altered by FN (Fig 10D). The level of vimentin expression was not altered by PE or FN (Bar Graph). Together, these data demonstrate that stimulation of α_1A_ARs increases expression of smooth muscle markers and MyoD in rat-1 cells, independently of cAMP.

## Discussion

This study is the first demonstration of the ability of an α_1A_-AR subtype to promote differentiation of a fibroblast into a SMC/myocyte-like cell. Further, it demonstrates that α_1A_-AR-induced cell cycle arrest and hypertrophy as well as differentiation is mediated through a mechanism dependent upon the increased expression of the critical cell cycle protein p27^kip1 ^and selective smooth muscle markers.

Stimulation of α_1A_-ARs promotes hypertrophy of cardiac myocytes [6]. Norepinephrine increases SMC hypertrophy, but not cell proliferation, through α_1A_-AR stimulation in uninjured aorta [8]. In HepG2 and M12 cells transfected with α_1A_-AR, PE stimulates cell scattering and inhibition of proliferation [15]. Similar observations have been made in CHO cells expressing α_1A_-ARs [17,18]. In the present study, PE inhibited cell proliferation, promoted hypertrophy and differentiation through stimulation of α_1A_-ARs in rat-1 cells expressing this subtype. Analysis of the effect of α_1A_-AR stimulation on cell cycle progression revealed that it inhibits the re-entry of cells from G_0_/G_1 _into the S phase of the cell cycle.

Next we attempted to define the mechanism mediating α_1A_-AR-induced cell cycle arrest, hypertrophy and differentiation of rat-1 cells. Previous studies have shown that in rat 1-cells expressing α_1A_-ARs, PE promotes increase in cAMP accumulation and PKA activation [12]. Since cAMP has been reported to inhibit growth in various cell types, including cells of fibroblastic origin [32], it seemed possible that cAMP mediates the inhibitory effect of PE on rat-1 cells proliferation. Stable cAMP analogs or adenylyl cyclase activation promoted cell cycle arrest similar to α_1A_-AR stimulation. However, activation of the cAMP/PKA pathway did not promote hypertrophy or differentiation of rat-1 cells. Moreover, inhibition of cAMP/PKA did not reverse the α_1A_-AR-induced inhibition of DNA synthesis. Therefore, α_1A_-AR-induced cell cycle arrest, hypertrophy and differentiation, is independent of the cAMP/PKA pathway in rat-1 cells. Recently, the cAMP pathway has been implicated in α_1A_-AR-induced cell cycle arrest in CHO cells expressing α_1A_-ARs [18]. The difference between these cell lines may be due to the inhibitory effect of PE on ERK in rat-1 cells [33] vs. PE-induced ERK activation in CHO cells [18]. In addition, the potential effect of α_1A_-AR stimulation on CHO cells hypertrophy and differentiation is not known. During our investigation of the possible mechanism by which PE induces cell cycle arrest, we observed that although both PE and FN inhibited DNA synthesis, these two agents produced remarkably different morphological changes in rat-1 cells. FN promoted a change in rat-1 cells to a small, shrinked, round shaped morphology whereas PE shifted the cells to a hypertrophied, enlarged and spindle shaped smooth muscle-like phenotype. This α_1A_-AR-induced morphological change was again independent of cAMP. Differences between PE and cAMP on different cell functions are reported in Table 3. EGF increases DNA synthesis in rat-1 fibroblasts [22] and slightly increased protein synthesis (our data). However, EGF was a far less potent hypertrophic agent than PE and did not change the morphology of rat-1 cells.

**Table 3:** Comparison of the effects of PE and FN on different cell parameters.

**Parameter**	**PE**	**FN**
cAMP production	↑	↑
Protein synthesis	↑	↓
DNA synthesis	↓	↓
Morphology	enlarged; spindle-shaped	shrunk; rounded
Differentiation markers	↑	↓
Cdk inhibitors	↑	↑

The arrows indicate a significant increase or decrease elicited by PE or FN in the corresponding cell parameter studied. These agents' stimulated cAMP production, decreased basal DNA synthesis, and up-regulated cyclin-dependent kinase inhibitors. PE and cAMP have opposite effects on protein synthesis, cell morphology and smooth muscle differentiation markers.

The different effects of α_1A_-AR stimulation and cAMP on rat-1 cell hypertrophy and morphology led us to explore the effects of PE and FN on different cell parameters associated with morphological changes, i.e. cell cycle regulators and smooth muscle cell differentiation markers. Recently, it has been reported that the Cdk inhibitor p27^kip1 ^mediates angiotensin II-induced cell cycle arrest and hypertrophy in cultured renal tubular cells [34], and vascular smooth muscle cells [20] and induces intestinal epithelial cell differentiation [35]. In the present study, stimulation of α_1A_-ARs increased the expression of p27^kip1 ^to a much greater extent than p21^cip1/waf1^, suggesting an important role for the former Cdk inhibitor in the action of α_1A_-AR stimulation. Interestingly, the time of upregulation of p27^kip1 ^by PE correlated closely with its effect on inhibition of DNA synthesis, which was maximal at 18 h. Depletion of p27^kip1 ^prevented both PE-induced upregulation of p27^kip1 ^and reversed PE-induced decrease in DNA synthesis. Therefore, p27^kip1 ^plays a central role as a mediator of α_1A_-AR-induced inhibition of DNA synthesis and probably hypertrophy and differentiation of rat-1 fibroblasts.

An important finding in the present study was that PE produced a change in the morphology of rat-1 cells characterized by an increase in cell size. Supporting this phenotypic and hypertrophic change was our demonstration that PE increased global protein synthesis and the expression of markers specific to smooth muscle cells such as smooth muscle actin, caldesmon, and myosin heavy chain. The protein levels of these smooth muscle cell markers were found to remain elevated for up to 48 h, consistent with the PE-induced morphological change that persisted for the same time period. Although stimulation of α_1A_-ARs shifted rat-1 fibroblasts to SMC/myocyte-like phenotype, it also increased the expression of the helix loop helix protein MyoD, a skeletal muscle-specific regulatory transcription factor [31]. Surprisingly, PE also caused an increase in pRb expression to a level similar to p27^kip1^. MyoD has been shown to interact with pRb and to promote muscle gene activation and cell cycle arrest [28,36]. Indeed, pRb has been found to contain a differentiation-promoting activity that is distinct from its cell-cycle progression functions [37]. More recent evidence has indicated an essential role for pRb in promoting functional synergism between MyoD and MEF2 proteins [38]. Although PE increased the expression of MyoD in our study, the rat-1 cells had a smooth muscle/myocyte-like phenotype. It has been reported that vascular smooth muscle cells can spontaneously adopt the skeletal muscle phenotype [31]. Therefore, it is possible that PE increases expression of MyoD in rat-1 cells during differentiation into myocyte-like cells. However, expression of MyoD is not sufficient for the coordinated program of skeletal myogenesis in smooth muscle cells [31].

PE-induced hypertrophy and differentiation of rat-1 cells was also associated with increased levels of cell cycle proteins pRb, cyclin D1, PCNA and Cdk2 that are important for G_1_/S phase progression [39]. These data indicate that stimulation of α_1A_-ARs promotes the transcriptional/translational activation of the machinery required for G_1_/S cell cycle progression. However, a simultaneous increase in Cdk inhibitors such as p27^kip1 ^prevented DNA synthesis. Elevation of p27^kip1 ^protein level alone is sufficient for induction of cell cycle arrest, independent of cyclins or Cdk level [40]. Surprisingly, PE also increased the protein level of Cdk4, which is normally not affected by mitogenic stimuli. Although the Cdk inhibitors bind to cyclin/Cdk complexes and reduce their activity, their interaction is probably much more complex [25,26]. Cdk inhibitors may paradoxically activate these kinases, particularly cyclin D/Cdk4, 6 complexes [26].

The mechanism by which stimulation of the α_1A_-AR promotes the up-regulation of Cdk inhibitors (p27^kip1^, p21^cip1/waf1^), smooth muscle cell markers, MyoD, and G_1_/S transition cell cycle proteins (pRb, cyclin D1, PCNA, Cdk2/4) leading to inhibition of proliferation and stimulation of hypertrophy and differentiation is not known. The cAMP/PKA pathway was excluded by our results as well as the ERK pathway [33]. The phosphatidylinositol 3-kinase and Akt/PKB pathway, a pro-survival/mitogenic and hypertrophic pathway is also unlikely to be involved, because PE does not stimulate this pathway in rat-1 cells [42]. Recently, a study on the genetic profiling of rat-1 fibroblasts expressing different subtypes of α-AR has shown that in cells expressing the α_1A_-AR, epinephrine (one hour stimulation) increased the gene expression of IL-6, gp-130 (an IL-6 high affinity receptor and signal transducer) and STAT-3 (an IL-6 activated transcription factor) [42]. Moreover, in cells expressing α_1A_-adrenergic receptor, epinephrine also increased IL-6 secretion and STAT-3 Ser^727 ^phosphorylation [42]. Therefore, it is possible that IL-6, gp130, and/or STAT-3 contributes to the upregulation of one or more of the cell cycle associated proteins and smooth muscle cell markers responsible for the cells arrest, hypertrophy and/or differentiation caused by α_1A_-AR activation in rat-1 cells.

With regard to functional significance *in vivo*, our model uses a transformed embryonic fibroblast cell line that expresses high levels of α_1A_-AR [12]. Therefore, the relevance of these results to fibroblasts in tissues remains to be determined. These features in our model may underlie the ability of PE to cause expression of SMC markers and MyoD through α_1A_-AR.

## Conclusions

This study demonstrates that stimulation of α_1A_-ARs in rat-1 cells promotes cell cycle arrest by increasing levels of Cdk inhibitors and promotes hypertrophy and differentiation into a phenotype having the characteristics of smooth muscle cells by a mechanism independent of cAMP or EGF. Moreover, cell cycle progression was blocked at G_1_/S transition without causing apoptosis, and this cycle arrest was critical for rat-1 cell hypertrophy and differentiation. Reducing p27^kip1 ^levels reversed α_1A_-AR-promoted inhibition of DNA synthesis. Furthermore, it shows that cell cycle arrest and differentiation are closely coordinated processes but temporally separable. Further studies are underway in our laboratory to characterize the signaling pathway(s) involved in α_1A_-AR-induced differentiation of fibroblasts to smooth muscle cells.

## Methods

### Materials

[Methyl-^3^H]thymidine (20 Ci/mmol) was purchased from NEN Life Science Products, Inc. (Boston, MA). L-[4,5-^3^H] leucine from Amercham Pharmacia Biotech. (Piscataway, NJ.). L-phenylephrine hydrochloride, penicillin, streptomycin, prazosin, propranolol, epidermal growth factor (EGF), and propidium iodide were obtained from Sigma (St. Louis, MO). Forskolin (FN), 8-cpt-cAMP and SQ 22536 were purchased from Calbiochem (La Jolla, CA). 2'-5'dideoxyadenosine and 2'-deoxyadenosine 3'-monophosphates were purchased from Biomol (Playmouth Meeting, PA). G418 sulfate from Invitrogen (Carlsbad, CA). Hanks' balanced salt solution and fetal bovine serum were from Mediatech, Inc. (Herndon, VA). Dulbecco's Modified Eagle's Medium (DMEM) and trypsin/EDTA were obtained from Life Technologies Inc. (Grand Island, N.Y).

### Rat-1 cells and culture conditions

Rat-1 cells transfected with bovine α_1A_-AR kindly provided to us by Drs. L. Allen, R. J. Lefkowitz, and M. G. Caron (Duke University), were maintained in DMEM supplemented with 10% (v/v) fetal bovine serum, 400 μg/ml G418 sulfate, 50 μg/ml streptomycin and 50 units/ml penicillin at 37°C in an humid atmosphere (5% CO2, 95% air). Cells were serially passaged upon reaching confluence, and all experiments were performed on subculture passages 5–15. Prior to all experiments, preconfluent cell cultures were serum-starved for 48 h.

### Measurement of DNA synthesis

Cells at a density of 150,000 cells/well were seeded in DMEM containing 10% FBS on 24-well plates until they reached 80–90% confluency and were serum-deprived for 48 h prior to the addition of agonists. Rat-1 cells, even after serum-deprivation for 2 days, exhibit a measurable level of [^3^H]thymidine incorporation. Inhibition of [^3^H]thymidine incorporation was used as a quantitative measure of reduction in DNA synthesis. Cells were serum-starved in the presence or absence of agonists for the indicated time period, and [^3^H]thymidine (0.5 μCi/ml) was added for the last 4 h of the incubation. This time period was chosen because it gave the maximum incorporation of [^3^H]thymidine into DNA after addition of agonists. The medium was then removed, and the cells were washed twice with phosphate-buffered saline and three times with 10% ice-cold trichloroacetic acid. Cells were kept in contact with trichloroacetic acid for 10 min during each wash. The precipitated material was dissolved in 1 M NaOH containing 0.1% SDS, and radioactivity was determined by liquid scintillation spectrometry.

### Measurement of protein synthesis

Rat-1 cells were seeded at a density of 150,000 cells/well in 24-well plates and just prior to confluency were serum-deprived for 48 h. Cells were rinsed with DMEM and 1 μCi/ml [^3^H]leucine was added for the last 4 h of each incubation with or without the indicated concentrations of the agonists. Cells were fixed and washed three times with ice-cold trichloroacetic acid (10%). The precipitated material was solubilized with 0.1 M NaOH and the incorporated radioactivity was counted by liquid spectrometry.

### Flow cytometry

Flow cytometry studies were performed to determine the effect of different concentrations of PE on cell cycle phases. Cells were subjected to flow cytometric DNA analysis as described [43] with some modifications. Briefly, rat-1 cells plated on 100 mm dishes were grown in DMEM containing 10% FBS until they reached 80–90% confluency. Preconfluent cell cultures were serum-starved for 48 h to stop the mitogenic effect of growth factors. The medium was aspirated and cells were incubated for 18 h with different concentrations of PE. Cells were trypsinized in 1 ml trypsin/EDTA for at least 5 min, and then the reaction was stopped by the addition of 1 ml of serum-containing DMEM. The samples were centrifuged at 1000 rpm for 5 min, washed three times in ice-cold PBS containing 1% bovine serum albumin by centrifugation at 1000 rpm for 5 min each, resuspended in 0.5 ml of the same solution, and then fixed with 1 ml of 70% ethanol (-20°C) added dropwise. The fixed cells were stored at 4°C until analyzed. Cells were then washed three times by centrifuging at 1000 rpm for 10 min and resuspended in 3 ml of the BSA buffer. The pellet was then finally resuspended in 1 ml of the BSA buffer to which 100 μg/ml of RNAse A was added to remove interfering RNA, and 5 μg/ml propidium iodide was added to stain DNA. Cells were incubated at 37°C for 10–15 min in the dark to facilitate staining. The cells were analyzed for DNA content using an Epics Profile Analyzer (Coulter Electronics) with an Argon laser emitting at 448 nm. Percentages of cells in various stages of the cell cycles were determined using a multi-cycle program (P. Rabinovitch, Phoenix Flow Systems). At least 10,000 cells per cycle were counted for each treatment.

### Cell viability

Preconfluent cultures were serum-starved for 48 h. Cells were incubated with different concentrations of PE, FN and 8-cpt-cAMP for 18 h, trypsinized with 1 ml trypsin/EDTA for 5 min and centrifuged for 10 min at room temperature. The pellet was resuspended in 1 ml plain DMEM. 10 μL of the suspension was mixed with 10 μL of 0.4% trypan blue in a 0.5 ml microtube. The total number of cells and the number of blue cells and the percentage of the viable cells in 10 μL of the trypan blue mixture was calculated as follows: % viable cells = [1-(blue cells/total cells)] × 100.

### Morphological study

Rat-1 cells resuspended in culture medium were seeded on six-well plates (Corning, N.Y.). Preconfluent cells were serum-deprived for 2 days, pre-incubated with inhibitor (s) for 30 min, and then stimulated with 5 μM PE and /or 1 μM FN for the indicated time. Cells were rinsed twice in PBS to remove non-adherent cells and then fixed for 10 min in [1:1] methanol-acetone mixture at room temperature. Cells were washed once in distilled water and stained in hematoxylin solution (Sigma, St. Louis, MO) for 15 min at room temperature. Cells were again washed three times in water, air dried and were finally observed under a phase contrast microscope. Images were captured and saved as TIFF files. For each condition, data were collected by random observation. Hypertrophic phenotype was defined as both enlarged, elongated and spindle-shaped whereas rat-1 fibroblasts are round and/or polygonal-shaped cells.

### Transfection procedures

To determine whether protein kinase A (PKA) mediates the antiproliferative effect of PE, rat-1 cells were transiently transfected with πLXX-PKI [1-31,24], a plasmid encoding for the PKA inhibitor, PKI (A generous gift from Dr. J. Avruch, Massachusetts General Hospital, Boston, MA), using Lipofectamine Plus (Life Technologies, Inc., Grand Island, N.Y.) according to the manufacturer's instructions. For p27^kip1 ^experiments, phosphorothionate oligonucleotides (ODN) (Life Technologies, Grand Island, N.Y.) were used. The antisense ODN sequence used in the experiments was 5'-CACTCTCACGTTTGACAT-3' (nuc 1–18 of rat p27 kip1); the sense ODN sequence was 5'-ATGTCAAACGTGAGAGTG. ODN transfection was performed with oligofectamine in Opti-MEM according to the manufacturer's protocol (Invitrogen, Carlsbad, CA). Cells were left in the transfection mixture for 48 h and then incubated for 24 h with or without agonists and harvested for Western blot, or labeled with [^3^H]thymidine to determine DNA synthesis.

### Preparation of cell lysates and western blot analysis

Cells were rinsed twice with PBS and lysed in ice-cold lysis buffer (1% Igepal CA-630, 25 mM HEPES pH 7.5, 50 mM NaCl, 50 mM NaF, 5 mM EDTA, 10 mg/ml aprotinin, 10 mg/ml leupeptin, and 5 mg/ml pepstatin A, 100 mM PMSF, 100 mM sodium orthovanadate, and 10 μM okadaic acid). Lysates were centrifuged at 4°C for 15 min in a microfuge at maximum speed, and the supernatant was collected for Western blot analysis. Equal amounts of protein were separated on denaturing SDS/polyacrylamide gel and transferred on nitrocellulose blots (Hybond-ECL; Amersham Life Sciences Inc., Arlington Heights, IL) by electrophoresis. Blots were blocked for 1 h in 5% nonfat dry milk in TBST, washed three times 5 min each in TBST and then incubated with the indicated specific primary antibody overnight at 4°C: p27^kip1^, p21^waf1/cip1^, pRb (Biosource International); caldesmon (smooth muscle), myosin (smooth muscle), α-smooth muscle actin, β-actin (Sigma Biosciences); MyoD, cyclin D1, Cdk 2, Cdk 4, PCNA, lamin A/C or vimentin (Santa Cruz Biotechnology). Following incubation, the membranes were washed three times 10 min each in TBST and incubated with a secondary antibody coupled to peroxidase for 1 h at room temperature. Then the membranes were washed three times 10 min each in TBST. Specific proteins were detected by enhanced chemiluminescence (ECL; Amersham Life Sciences Inc.) according to the manufacturer's instructions and analyzed with an Alpha Innotech Fluorochem imaging system (Packard Canberra). A lysate from cultured aortic vascular smooth muscle cell (VSMC) was used as a control for the expression of smooth muscle markers.

### Statistical analysis

The basal values of incorporation of [^3^H]thymidine and/or leucine were variable in different batches of cells. However, the effect of various agents on the incorporation of [^3^H]thymidine and [^3^H]leucine in rat-1 cells was consistent within each batch of cells. The results are expressed as mean ± SEM. The data were analyzed by one-way analysis of variance; the Newman-Keuls multiple range test was applied to determine the differences among multiple groups, the unpaired Student's t-test was applied to determine the difference between two groups. The null hypothesis was rejected at p < 0.05. The protein level were estimated by densitometric analysis of the Western blots and performed on the indicated number of blots using NIH Image software, and expressed as a percentage of the control, arbitrarily chosen as 100%.

## Abbreviations

AR, adrenergic receptor; Cdk, cyclin-dependent kinase; FN, forskolin; MHC, myosin heavy chain; PCNA, proliferating cell nuclear antigen; PE, phenylephrine; pRb, retinoblastoma protein; SMC, smooth muscle cell

## Authors contributions

AES performed thymidine/leucine incorporation, morphological studies, cell viability, flow cytometry and Western blot analysis. JHP carried out the antisense design, transfection experiments, statistical analysis and some thymidine/leucine incorporation and Western blot analysis. KUM conceived of the study, and participated in its design and coordination. All authors read and approved the final manuscript.
